# Editorial: Applied biophysics of the tooth and dental materials

**DOI:** 10.3389/fdmed.2023.1258974

**Published:** 2023-07-31

**Authors:** Sebastian Aguayo, Nelson P. Barrera, Laurent Bozec

**Affiliations:** ^1^ Dentistry School, Faculty of Medicine, Pontificia Universidad Católica de Chile, Santiago, Chile; ^2^ Institute for Biological and Medical Engineering, Schools of Engineering, Medicine and Biological Sciences, Pontificia Universidad Católica de Chile, Santiago, Chile; ^3^ Department of Physiology, Faculty of Biological Sciences, Pontificia Universidad Católica de Chile, Santiago, Chile; ^4^ Faculty of Dentistry, University of Toronto, Toronto, ON, Canada

**Keywords:** biophysics, microfabrication, mineralization, dental research, interdiscipinary research

**Editorial on the Research Topic** Applied biophysics of the tooth and dental materials

In recent years, the field of dental sciences has witnessed remarkable advancements in the application of interdisciplinary approaches that have revolutionized craniofacial research in areas including dental biomaterials, cariology, diagnostics, and disease treatment. Within this context, biophysics—a multidisciplinary field that combines principles from biology and physics to study biological principles—offers a unique perspective to investigate and comprehend the intricate mechanisms underlying oral health and disease such as the dynamics of tooth demineralization-remineralization, the development and optimization of novel bio-active materials, and the interaction between the oral microbiome, biomaterials, and tissues, amongst others. Therefore, in this Research Topic, we aimed to highlight how applied biophysical methods and approaches are currently being employed in dental research as well as their potential to improve oral healthcare practices from bench to bedside.

Our current *Applied Biophysics of the Tooth and Dental Materials* Research Topic is comprised of three original articles and one review article that feature the use of advanced microscopy, quantitative atomic force microscopy (AFM), elemental analyses, ^19^F magic angle spinning nuclear magnetic resonance (MAS-NMR) spectroscopy, and microfabrication approaches, as tools to answer relevant dental research questions that cannot be explored solely from a biological standpoint. In this regard, the original contribution by Pattem et al. utilized advanced AFM-based quantitative imaging to explore dental erosion and the structure-property relationships between dietary acid concentration and pH, and their influence on the nano-textural and nano-mechanical properties of human enamel and dentine in an ex-vivo model. Dietary acid exposure was found to induce important mechanical and morphological changes in both tissues at the nanoscale, and interestingly, authors found that dentin was less susceptible to acid erosion in their model possibly due to the protective effect of the dentinal collagen matrix.

Complimentarily, the work by Li et al. and Ferizoli et al. investigated the remineralizing effect of a novel amelogenin peptide and fluoride, respectively, on previously demineralized surfaces. Li et al. monitored remineralization through a range of biophysical and interdisciplinary approaches including micro-CT, polarized light microscopy, scanning electron microscopy, x-ray-micro-diffractometry, and isothermal titration calorimetry. Results showed that peptide treatment of a demineralized enamel surface yielded mineral deposition in the form of hydroxyapatite (HAP) crystals and could be a promising alternative for future conservative approaches against enamel caries lesions. On the other hand, Ferizoli et al. monitored the remineralization of HAP surfaces by fluoride using MAS-NMR spectroscopy and compared it to previous investigations on enamel substrates. The authors found that, at concentrations of 50 ppm and above, calcium fluoride was formed on the HAP surface; however, at concentrations below 50 ppm, fluoridated apatite was produced. As a result, HAP disks can be used as an effective enamel analog for use in the in-vitro investigation of the role of fluoride in enamel mineralization.

Finally, Tiozzo-Lyon et al. contributed to the Research Topic with a review of the current and cutting-edge microfabrication approaches being utilized in both basic dental research and clinics. Although microfabrication techniques such as microfluidics, organs-on-a-chip, and photolithography have been around for some time, their application in the oral research field is still quite incipient and efforts need to be made to showcase their utility. This review highlighted how researchers across the globe are utilizing microfabrication to answer pressing research questions in dental biomaterials, oral biology, and periodontal regeneration, among others. Furthermore, microfabrication has quickly been gaining traction in clinics and soon may be available to support the diagnosis and treatment of prevalent dental and craniofacial pathologies.

Overall, we hope that this Research Topic aids in promoting the visibility of the broad range of biophysics-dentistry collaborations currently taking place across the globe and encourages engagement between researchers from basic sciences and dental clinicians. Towards the future, we expect that the trend of looking at dentistry-related issues through a biophysical lens continues to unravel key questions regarding the complexity of oral tissues, microbiomes, and restorative materials and procedures.

